# Study of the Intrinsic Fluorescence of a Highly Branched Cationic Dendrimer, Poly(Ethyleneimine) (PEI)

**DOI:** 10.3390/molecules24203690

**Published:** 2019-10-14

**Authors:** Viktória Tóth, Péter Hermann, Dániel Végh, Tivadar Zelles, Zoltán Géczi

**Affiliations:** 1 Department of Prosthodontics, Semmelweis University, Szentkirályi u. 47, 1088 Budapest, Hungary; toth.viktoria2@dent.semmelweis-univ.hu (V.T.); hermann.peter@dent.semmelweis-univ.hu (P.H.); vegh.daniel@dent.semmelweis-univ.hu (D.V.); 2 Department of Oral Biology, Semmelweis University, Nagyvárad tér 4, 1089 Budapest, Hungary; zelles.tivadar@dent.semmelweis-univ.hu

**Keywords:** biopolymers, biodegradability, intrinsic fluorescence, polyethyleneimine, dendrimer, molecular weight

## Abstract

Poly(ethyleneimine) (PEI) is a weakly basic, synthetic, polycationic polymer, due to the presence of primary, secondary, and tertiary amino groups. The amino groups are responsible for the variety of applications of PEI (e.g., transfection, bioimaging, solar cell, etc.). Our study presents some new and reproducible methods for the quantification of molecular or mass concentration of highly branched PEI of different molecular weights (800–2000–25,000–750,000 MW PEI). In the course of the direct method, spectrophotometry and fluorometry were applied to determine the absorption and fluorescence of PEI dilution series. An increase in the MW at the same concentration produces a higher count number because of the higher number of amino groups in PEI molecules. The character of increment in fluorescence intensity is essentially different in the case of mass concentrations and molar concentrations. The increment of the fluorescence intensity related to the molar concentration is non-linear. In the case of mass concentration, the slope is linear. Moreover, their fluorescence is enhanced with the decrease in pH values. The spectrophotometry is a reliable method for measuring the quantity of PEI molecules in solution. Our data help in recognizing the detailed properties of PEI in dendrimer research.

## 1. Introduction

The reason for the theme selection of this publication is the present and future central role of poly(ethyleneimine) (PEI) in Biomedical Sciences and biochemical engineering. Reccently, PEI is the most commonly utilized non-viral synthetic vector in gene delivery apart from viral transduction (‘golden standard’) [1]. Not only nucleic acids but also almost all molecules (e.g., antibiotics and anti-cancer drugs) can be delivered into cells, so far as they are bound to PEI. There is some promising research for using PEI in the field of solar panels and bioimaging [2,3]. These diverse applications of PEI are a consequence of its chemical structure. Due to its numerous positive charges, it can easily enter the cell membrane via endocytosis, which makes it an optimal agent for gene transfer [4]. PEI is a polycationic polymer having linear and branched forms with different molecular weights [5]. The most often used form is the highly branched form which gives the molecule a spherical form in solution [6]. The most often applied form in gene delivery is the 25,000 molecular weight (MW) PEI which possesses more than 50 primer amino groups in the end position of its branched chains. Owing to the high number of positive amino groups, PEI has a great affinity to negatively charged membranes. The same characteristic feature of PEI is responsible for the formation of complexes, composites, therefore, hence its clinical application. PEI is an effective antimicrobial molecule which is antibacterial and antifungal agent as well. Due to its special antimicrobial mechanism, there is no antimicrobial resistance against it. Our laboratory synthesized a novel antimicrobial composite, Ag–PEI–polylactic acid (PLA) utilizing its antimicrobial activity [7]. An additional role is that it can be applied as a carrier for osteoinductive growth factors (e.g., bone morphogenetic proteins) to alveolar bone. Poly(ethyleneimine) in higher concentrations induces cell membrane disorganization, and mitochondrial disruption over time, resulting in apoptosis.

The biological and chemical efficiency of PEI can be evaluated with various methods e.g., microbiological assays, pharmacological assays, spectrophotometry, etc. Generally, the quantitative determination of PEI is carried out by UV-Vis methods which are fairly time consuming and costly.

Our latest publication requires the quantitative determination of PEI from the released materials [7]. In these samples, the PEI may be bounded and unbounded. The bonds in the PEI complexes are mainly hydrogen or electrostatic bonds. The possibility of the breaking of these bindings makes the determination of PEI uncertain, that is why we measured the fluorescence of PEI (intrinsic). This intrinsic, non-traditional weak fluorescence [8] of dendrimers that do not possess classical fluorophores is an increasingly studied phenomenon recently [9]. Fluorescence is the result of electron−hole recombination on delocalized states so that emission wavelength depends on excitation wavelength. This inherent fluorescence characterizing polyethyleneimines can be interpreted by the formation of amine rich nanocluster and electron-hole recombination processes [10].

The aim was to find the relation between the molecular weight, concentration, pH, and the fluorescence intensity of PEI. Besides the fluorescent measurement, we planned to specify the absorbance properties of PEI solutions.

## 2. Results

### 2.1. The Measurement of Fluorescence of PEI Dilution Series with Different Molecular Weights

**Figure 1:**
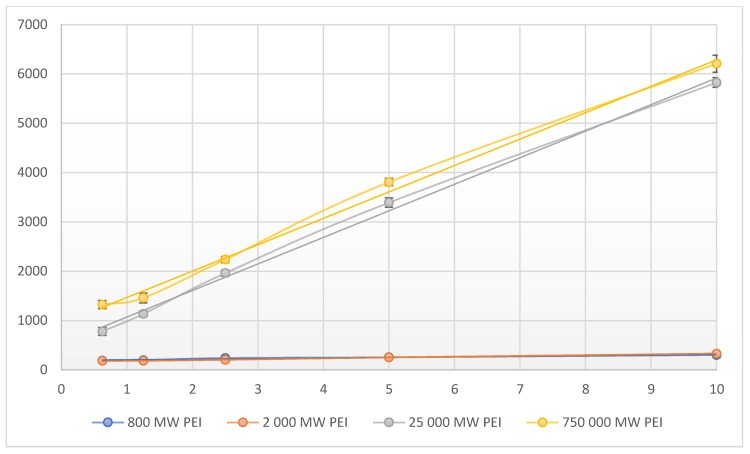
Fluorescent intensity related to mass concentration (0.625–1.25–2.5–5–10 mg/mL (*w/v*)) of PEI with different molecular weights (0.8–2–25–750 kPEI) dilution series. Technical parameters: λ_ex_ = 280 nm; λ_em_ = 290–720 nm; scan speed = 1200 nm/min; PMT voltage = 700 V. Maximal emission: λ_em_ = 560nm; pH ≈ 10.6. The standard 3.5 mL quartz cuvette were OD: 12.5 mm × 12.5 mm × 45 mm; wall thickness: 1.25 mm with 10 mm light path and four polished sides. The volume of all the samples was 1 mL. Technical parameters of the assay—sample number: 5/measuring point; temperature: 23 °C.

**Figure 2:**
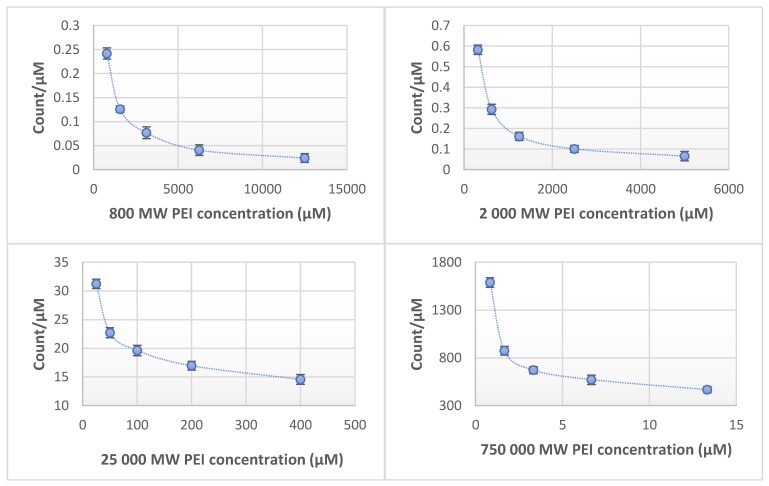
Fluorescent intensity related to molar concentration (count/µM) of PEI with different molecular weights (0.8–2–25–750 kPEI) dilution series. Technical parameters: λ_ex_ = 280 nm; λ_em_ = 290–720 nm; scan speed = 1200 nm/min; PMT voltage = 700 V. Maximal emission: λ_em_ = 560 nm; Ph ≈ 10.6. The standard 3.5 mL quartz cuvette were OD: 12.5 mm × 12.5 mm × 45 mm; wall thickness: 1.25 mm with 10 mm light path and four polished sides. The volume of all the samples was 1 mL. Technical parameters of the assay—sample number: 5/measuring point; temperature: 23 °C.

The fluorescence of PEI dilution series (0.625–1.25–2.5–5–10 mg/mL (*w/v*)) were measured in different molecular weights (0.8–2–25–750 kPEI). In every case, the increase of fluorescence was linear and could be observed with increasing concentrations. In the case of 0.8 kPEI and 2 kPEI, the count rate increased slightly while the count rates of 25 kPEI and 750 kPEI increased in higher proportions (Figure 1). Based on the measurements of the linear correlation coefficients the relationship is strong (800 MW PEI = 0.9634; 2000 MW PEI = 0.9965; 25,000 MW PEI = 0.9983; 750,000 MW PEI = 0.9979). It was detected that the increase of molar concentration results in a non-linear fluorescent increment (Figure 2). The limit of detection (LOD) for each molecular weights of PEI: 0.8 kPEI 0.625 mg/mL (measured); 2 kPEI 0.625 mg/mL (measured); 25 kPEI 0.229 mg/mL (extrapolated from data); 750 kPEI 0.220 mg/mL (extrapolated from data).

### 2.2. The pH Measurements of PEI Dilution Series

**Figure 3:**
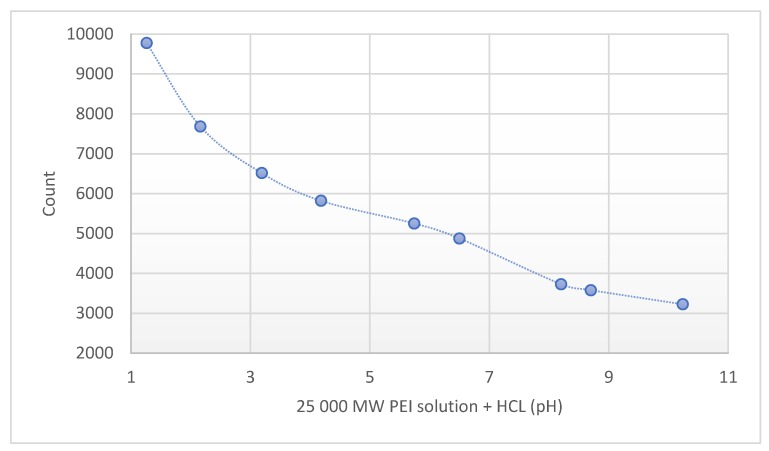
Fluorescence intensity of 25 kPEI solution (10 mg/mL) at different pH. Technical parameters: λ_ex_ = 280 nm; λ_em_ = 290–720 nm; scan speed = 1200 nm/min; PMT voltage = 700 V. Maximal emission: λ_em_ = 560 nm. The standard 3.5 mL quartz cuvette were OD: 12.5 mm × 12.5 mm × 45 mm; wall thickness: 1.25 mm with 10 mm light path and four polished sides. The volume of all the samples was 1 mL.

The pH of the different molecular weight of PEI solutions (0.8–2–25–750 kPEI) was pH ≈ 10.6 at the same concentration. Lower pH values correlate with higher fluorescence intensity (Figure 3).

### 2.3. The Measurement of Absorbance of PEI

**Figure 4:**
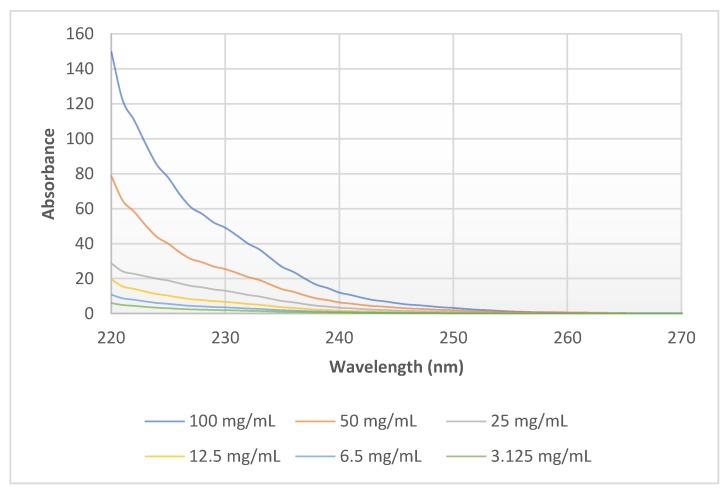
Absorbance spectra of 25 kPEI dilution series (100–50–25–12.5–6.25–3.125 mg/mL) in DW. UV-Vis analysis program was applied (λ = 220–750 nm) with 3 µL drop of solution after calibration with DW at room temperature (23 °C).

**Figure 5:**
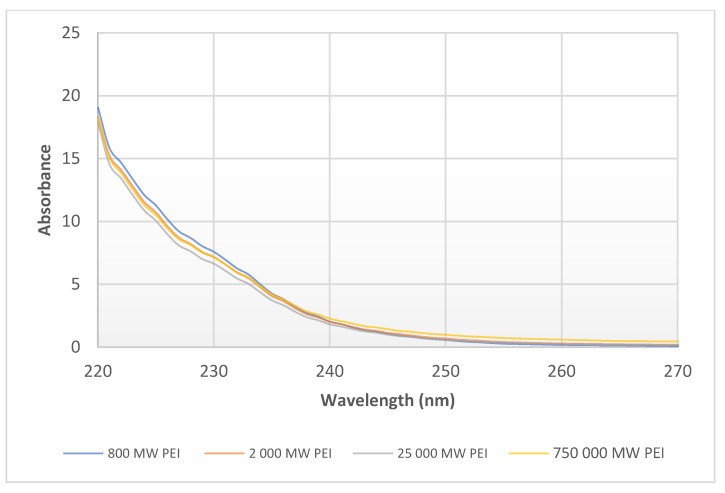
Absorbance spectra of 1% 0.8–2–25–750 kPEI samples in DW. UV-Vis analysis program was applied (λ = 220–750 nm) with 3 µL drop of solution after calibration with DW at room temperature (23 °C).

In the case of PEI dilutions (3.125–6.25–12.5–25–50–100 mg/mL (*w/v*)) the maximum of absorbance was at 220 nm in each sample. The growth in absorbance at 220 nm is directly proportional to change in concentration (Figure 4). There was no difference in the absorbance spectra at the same concentration (1%) of the different molecular weights of 0.8–2–25–750 kPEI (Figure 5).

## 3. Discussion

Some dendrimers such as poly(amino ester), poly(amidoamine) (PAMAM), and poly(ethyleneimine) can produce fluorescence emission, known as intrinsic-fluorescent emission phenomenon [11]. The basic understanding of intrinsic fluorescence was gained from the quantum chemical research of the PAMAM [12]. This fluorescent activity of PEI can be multiplied with the binding of certain substances, e.g., nucleic acids and metal ions [13]. The fluorescence measurement of the “native” PEI in solution is reliable if it does not contain other fluorescent molecules and the emission of PEI is not influenced (e.g., by quenching). In several cases, there is a need to determine the quantity of PEI in complexes as well. Similarly, the quantitative determination of PEI is inevitable during the planning phase of complex synthesis which aims to deliver materials into cells or modify the physical parameters of the complex for example.

However, the quantitative determination of “native” PEI from polymer-complexes or composites is difficult. It is important because the release of PEI from the composites basically determines the antimicrobial activity, the toxicity, and, consequentially, the clinical application of the composite.

The following parameters describe the properties of dendrimers, including PEI:

- The molecular weight is the primarily accountable property of the given polymers. It is especially true for the PEI, whose structure is built up according to so-called ‘generations’, like layers of an onion (onion-like structure). In every additional shell, the number of terminal amino groups may redouble. This is the reason why the number of reactive amino groups increases gradually. From generation to generation the synthesis increases in difficulty and cost. After the fifth generation, the branches may lean back and create intermolecular connections.
- The number of amino groups and their degree of protonation fundamentally determines the properties of PEI (transfection activity, complex formation, antimicrobial efficacy, fluorescence, etc.) [1,14].
- The pH of the solution basically determines the degree of protonation of amino groups [15]. The primary- and tertiary-amine groups’ protonation mechanism can explain the higher fluorescence emission phenomena of PEI at low pH.

The intensity of intrinsic fluorescence increases with the molecular weight. This relationship is not linear (data presented). The results show (Figure 2) that in the case of every molecular weight the lower molecular concentrations have higher count/µM rate than the higher concentrations. It allows us to make the hypothesis that there is an intrinsic inhibition in the case of higher concentrations.

The relation between the basic properties of PEI and the intrinsic fluorescence is poorly discussed in the literature, due to the practical challenges of investigating this relationship. In this publication, we are focusing on the properties of the most often used highly branched forms of PEI and not discussing the linear forms. The spectrophotometry, including the measurement of fluorescence, is one of the most suitable and easier method for the recognition of the properties of PEI molecules. This method includes the measurement of absorption and fluorescence of PEI.

Besides the most commonly used 25 kPEI, we determined the fluorescence of the 0.8–2–750 kPEI branched PEI solutions as well. Based on the results, it can be stated that not only the concentration of solutes but also the molecular weight of PEI can modify the intensity of the fluorescence. At a given MW the intensity of fluorescence depends on the mass concentration (mg/mL) linearly (Figure 1). While the fluorescence intensity strongly depends on the MW (hydrodynamic size, generations). The rate of increment was significantly greater in the case of 25 k- and 750 kPEI than 0.8 k- and 2 kPEI solutions. Between the different MW PEI at same concentrations, there is a lack proportion in the fluorescence intensity (e.g., 2000–25,000 MW). Besides the mass, molar concentration, and the MW we also studied the pH-dependence of the PEI fluorescence emission. According to Lourdes Pastor-Pérez et al. [16] the fluorescent intensity of polyethyleneimine polymers is heavily pH-dependent so we determined the pH-dependence fluorescence of the most often applied form of branched 25 kPEI as well.

The explanation of these results that the higher molecular weight PEI molecules have relatively more amino groups. The reason for this phenomenon is due to the free amino groups which are responsible for the fluorescence [17]. Having more free amino groups on the molecules means the count rate will be higher. The molar concentration of the PEI influences the intrinsic fluorescence. Consequently, an exact quantification of molecular or mass concentration by fluorescent intensities requires both molecular weight and pH to be controlled. There is another characteristic that the increment of the fluorescent intensity related to molar concentration (count/µM) is not linear (Figure 2) originated by a highly branched structure, (long branches, number of generations, and spherical effects). Owing to this hyperbranched structure the relationship between the increase in MW and the increment in the number of groups responsible for fluorescence is not linear.

The efficiency of PEI (antimicrobial and transfection effect) is based on the number of amino groups [18,19]. Based on this correlation, we can conclude that the efficacy of PEI can be determined indirectly from the fluorescence rate in the presence of the necessary measurement conditions. The value of absorbance is influenced by the concentration of solutes in solutions (Figure 4). The advantage of the absorbance measurement that it is not influenced by the molecular weights of PEI contrary to the fluorescent method (Figure 5). However, the disadvantage is that in the measurement of absorption the detection limit is higher. Therefore, both methods are suitable for the quantitative detection of PEI under controlled circumstances (e.g., pH is set, MW is known, etc.).

Our results clearly show, there is no linear relation between the molar concentration and the intensity of fluorescence. The connection between the intensity of fluorescence and the degree of the protonation of PEI request further analysis.

## 4. Materials and Methods

### 4.1. Materials

High-branched polyethyleneimine (PEI) with molecular weights of 750,000 MW (750 kPEI; Mn: 750,000 g mol^−1^, M_w_/M_n_ = 12.5), 25,000 MW (25 kPEI; Mn: 25,000 g∙mol^−1^, M_w_/M_n_ = 2.5), 2000 MW (2 kPEI; Mn: 2.000 g mol^−1^, M_w_/M_n_ = 1.11), 800 MW (0.8 kPEI; Mn: 800 g∙mol^−1^, M_w_/M_n_ = 1.33), hydrochloric acid, were purchased from Sigma-Aldrich Chemie, Germany. Deionized and ultrapure water (Merck, Germany) were used throughout the study; whose resistivity was ≥18 MΩ cm.

### 4.2. Fluorescence Measurements of PEI Dilution Series

The fluorescence intensity of 25 kPEI dilutions were determined using a Hitachi F-4500 FL Spectrophotometer. The dilution series were made (0.625–1.25–2.5–5–10 mg/mL (*w/v*)) from different molecular weight of PEI, 0.8–2–25–750 kPEI. The pH was 10.6 for each sample. The PEI was dissolved with mild shaking in Eppendorf tubes with distilled water for 4 h. The measurements were applied with the following parameters:


**Measurement type:**

**Wavelength Scan**
Scan mode:EmissionData mode:FluorescenceEX WL:280.0 nmEM Start WL:290.0 nmEM End WL:720.0 nmScan speed:1200 nm/minDelay:0 sEX Slit:5.0 nmEM Slit:5.0 nmPMT Voltage:700 VResponse:Auto

### 4.3. The pH and Fluorescent Measurements of 25 kPEI Dilution Series

The pH determination of PEI solutions in DW (0.8–2–25–750 kPEI MW) was carried out. The initial concentration (10 mg/mL) was used in the case of every molecular weight. The pH was measured with the pH meter, Hanna Piccolo Plus P9565-1EA from Sigma-Aldrich.

In the case of 25 kPEI, the fluorescent activity was measured at different pH values. The pH was set with hydrochloric acid. Each pH measurements were carried out in different samples supplemented until the same volume, therefore the possible measurement deviation derived from dilution was eliminated.

### 4.4. Absorption Measurements of PEI Dilution Series

For the measurement of absorption, the DeNovix DS-11 FX Spectrophotometer/Fluorometer device was used. The UV-Vis analysis program was applied (220–750 nm wavelength) with 3 µL drop of solution. The calibration of the device was carried out with distilled water and then the absorption spectra of 25 kPEI (10–5–2.5–1.25–0.625–0.3175 mg/mL) dilution series were measured. The absorbance spectra were determined in the case of 1% dilutions of 0.8–2–25–750 kPEI. The PEI was dissolved with mild shaking in Eppendorf tubes in distilled water for 4 h.

## 5. Conclusions

The simple and fast spectrofluorometric method was applied to study the properties of intrinsic fluorescence of PEI. In laboratory practice, the most commonly used dimension is the mg/mL. It is suggested to use also the molar concentration at the higher MW of PEI. The character of increment in fluorescence intensity is essentially different in the case of mass concentrations and molar concentrations. Change in fluorescence intensity in relation to the molar concentration is non-linear, while the relationship is linear in the case of mass concentration. Fluorescence intensity of PEI depends upon the pH of its solvent, as a decrease in pH will increase fluorescence, while maintaining PEI concentration. As such experimental conditions must be carefully controlled to ensure accurate results.
